# Know DBS: patient perceptions and knowledge of deep brain stimulation in Parkinson’s disease

**DOI:** 10.1177/17562864241233038

**Published:** 2024-03-06

**Authors:** Meagen Salinas, Umar Yazdani, Austin Oblack, Bradley McDaniels, Nida Ahmed, Bilal Haque, Nader Pouratian, Shilpa Chitnis

**Affiliations:** Department of Neurology, University of Texas Southwestern Medical Center, 5323 Harry Hines Blvd, Dallas, TX 75390, USA; Neurology Section, VA North Texas Health Care System, Dallas, TX, USA; Department of Neurology, University of Texas Southwestern Medical Center, Dallas, TX, USA; Department of Neurology, University of Texas Southwestern Medical Center, Dallas, TX, USA; Department of Rehabilitation and Health Services, University of North Texas, Denton, TX, USA; Department of Neurology, University of Texas Southwestern Medical Center, Dallas, TX, USA; Department of Neurology, University of Texas Southwestern Medical Center, Dallas, TX, USA; Department of Neurosurgery, University of Texas Southwestern Medical Center, Dallas, TX, USA; Department of Neurology, University of Texas Southwestern Medical Center, Dallas, TX, USA

**Keywords:** deep brain stimulation, health educations, Parkinson’s disease, patient education, questionnaire design

## Abstract

**Introduction:**

Deep brain stimulation (DBS) is an established therapy for Parkinson’s disease (PD) that can significantly improve motor symptoms and quality of life. Despite its effectiveness, little is known about patient perceptions of DBS.

**Objectives:**

To evaluate patient perceptions of DBS for PD, focusing on understanding, satisfaction, and factors influencing their outlook. This study aims to enhance patient education and counseling by identifying key determinants of patient perceptions.

**Design:**

A patient survey.

**Methods:**

We surveyed 77 PD patients who had undergone DBS at multiple centers using a comprehensive questionnaire. The questionnaire included questions on demographic information, disease history, and detailed understanding about the indications for DBS, side effects, outlook, and other common misconceptions. We summarize data using measures of central tendency and dispersion appropriate to the data type (categorical, continuous, proportional) and model relationships among variables using fractional and linear regression methods.

**Results:**

Participants had a median age of 66 years, were predominantly male (66%), Caucasian (90%), well-educated (79% with at least college degrees), and had a disease duration of greater than 5 years (97%). They conveyed good understanding of the signs and symptoms addressed by DBS across the motor and non-motor domains and associated side effects. Regression analysis identified age, disease duration, and education level as key determinants of patient understanding and outlook of DBS.

**Conclusion:**

Our study provides a detailed understanding of patient perceptions of DBS for PD, including the benefits, challenges, and misconceptions. Our findings underscore the importance of identifying the causes of disparities in patient knowledge and perceptions regarding DBS to tailor patient counseling and ensure optimal treatment outcomes.

Parkinson’s disease (PD) is a chronic neurodegenerative disorder characterized by motor symptoms such as bradykinesia, rigidity, tremor, and postural instability. While pharmacological interventions play a central role in managing PD symptoms, their efficacy may diminish over time and can lead to motor and behavioral complications. Deep brain stimulation (DBS) has emerged as an effective therapeutic option for PD, providing significant symptom relief and improving quality of life for patients.^1^

DBS involves the surgical implantation of electrodes into specific brain regions, followed by the delivery of electrical impulses to modulate abnormal neural activity. The subthalamic nucleus and the globus pallidus internus (GPi) are the most commonly targeted areas for PD DBS, yielding substantial improvements in motor symptoms and medication reduction.^2,3^ However, outcomes are attenuated by disease characteristics and burden. Mathers *et al*.^4^ found that factors such as age, depression, apathy, levodopa response, and time since disease onset may influence post-DBS quality of life outcomes. Rizzone *et al*.^5^ investigated risk factors for disability development with DBS and found that a higher baseline Unified Parkinson’s Disease Rating Scale (UPDRS)-III axial subscore, late-onset PD, and the presence of rapid eye movement (REM) sleep behavior disorder were associated with an increased risk.

While the clinical efficacy of DBS for PD has been well-documented, understanding patient perceptions and experiences is crucial for comprehensive treatment evaluation and patient-centered care.^6^ Patient perceptions encompass a range of aspects, including treatment expectations, satisfaction with outcomes, perceived drawbacks, and overall quality of life improvements. This distinguishes perception from knowledge, which we view as a determinant, or influential factor, of patient perceptions, and in fact explicitly model it as such (see Data analysis section under Methods).

Several studies have explored patient perceptions of DBS for PD, shedding light on various dimensions of this treatment modality. Cabrera *et al*.^7^ explored the role of sociodemographic factors in DBS decision-making and found that patient satisfaction with DBS is associated with earlier acceptance of the intervention. On the other hand, reasons for not agreeing to earlier DBS included not exhausting other medication alternatives and managing symptoms effectively with current medication. Interestingly, male patients have been found to be more likely to accept DBS earlier in the disease course compared to female patients.^8^

Other studies have focused on healthcare professionals and patient knowledge. Cabrera *et al*.^9^ highlighted that unclear patient expectations can influence neurologists to not refer patients for DBS. Rossi *et al*.^10^ discovered that PD patients may have negative perceptions of DBS outcomes due to unfulfilled expectations, and Prasad *et al*.^11^ suggested that patients often have inadequate and incorrect knowledge about DBS, leading to unrealistic expectations and lower satisfaction. In these studies, education, socioeconomic status, and disease severity were found to influence awareness of DBS.

Despite these valuable contributions, a comprehensive analysis of patient perceptions of DBS for PD, encompassing a wide range of factors, is still needed. This manuscript aims to address this gap by presenting a detailed examination of patient perceptions of DBS for PD, incorporating demographic information, disease history, treatment expectations, outcomes, side effects, and overall satisfaction. In doing so, we seek to rigorously explore the generalizability of the aforementioned findings as well as the relationships among these influential factors of patient perceptions of DBS in a relevant population. By capturing the multifaceted nature of patient experiences, we can gain valuable insights to improve clinical decision-making, patient counseling, and postoperative care.

## Methods

### Recruitment and participants

Participants were recruited from the Dallas Area Parkinsonism Society, Parkinson Voice Project, and the Aston Clinic Movement Disorders Clinic at UT Southwestern Medical Center (Dallas, TX, USA). Inclusion criteria stipulated that they be: English speaking, at least 18 years of age, carry a prior diagnosis of idiopathic PD, and cognitively competent to complete the survey. The exclusion criteria encompassed prisoners, non-English speakers, patients under the age of 18, and persons living farther than 150 miles from the Dallas–Fort Worth Metroplex area.

A link to the study was provided at the three recruitment venues for participants who self-identified as satisfying the inclusion/exclusion criteria to access. Seventy-seven participants completed the survey and were included in the analysis. Inspection of the data in the survey did not identify any who were in violation of these criteria.

### Survey

The survey was generated using RedCap and delivered to study participants electronically. It consisted of a 43-point questionnaire with integer (for age), categorical (for demographic questions), and binary yes/no or true/false (for questions querying patient perceptions or knowledge of DBS indications, outcomes, and possible side effects) responses. The survey was modeled after a prior questionnaire^6^ exploring patient perceptions of PD and the questions were drafted by Movement Disorders specialists involved in the pre- and post-surgical evaluation and management of DBS patients.

We did not collect any additional personal or health identifiable information and survey responses were stored electronically in a password-protected system accessible only by the study investigators.

### Data analysis

Survey data were analyzed in R (R Core Team, R Foundation for Statistical Computing, Vienna, Austria). No missing data were imputed. Demographic characteristics were summarized by median and interquartile range or by percent breakdown within each category. To investigate the relationship between demographic variables and patient knowledge of DBS, we used fractional regression as implemented by the *frm* package^12^ with the fraction of correct responses to factual questions about DBS as the dependent variable and demographic variables including age, disease duration, gender, education level, and race as the independent variables. Similarly, to investigate the relationship between patient perception of DBS and demographic variables, we modeled the fraction of affirmative responses to the questions/statements ‘Do you believe the outcomes of DBS show similar efficacy to the best medications/pharmacological management?’, ‘Do you believe DBS will alter the natural course of my PD?’, and ‘DBS programming has no known side effects’ as the dependent variable and the aforementioned demographic variables and number of correct responses to factual DBS questions as the independent variables. Model coefficients are presented as odds ratios (ORs) for interpretability, with only those parameters with *p* values less than 0.05 considered statistically significant. Linear relationships between certain pairs of variables were investigated by linear regression models. A stripchart was used to convey the distribution of the number of responses conveying a positive outlook of DBS stratified by educational level since the number of participants in each category affected the display of a boxplot.

## Results

### Patient characteristics

Seventy-seven participants completed the survey and all reported they had DBS. Their median age was 66, 34% were female, 90% identified as Caucasian, and most (79%) had at least graduated college. The majority of patients also reported a disease duration of greater than 5 years (97%). These characteristics are depicted in Table 1.

**Table 1.** Patient characteristics.

Characteristic	*N* = 77^a^
Age	66 (62, 73)
Gender	
Female	26 (34%)
Male	51 (66%)
Disease duration	
4–5 years	2 (2.6%)
More than 5 years	75 (97%)
Education	
A few years of college	7 (9.1%)
Graduated college	35 (45%)
High school diploma	8 (10%)
Post graduate education	26 (34%)
Some education but did not graduate from high school	1 (1.3%)
Race	
African American	2 (2.6%)
Asian or Pacific Islander	3 (3.9%)
Caucasian (White)	69 (90%)
Prefer not to say	3 (3.9%)
Deep-brain stimulation	
Yes	77 (100%)

a Median (IQR); *n* (%).

IQR, inter-quartile range; *N*, number.

### Landscape of patient knowledge and perceptions of DBS

**Figure 1.**
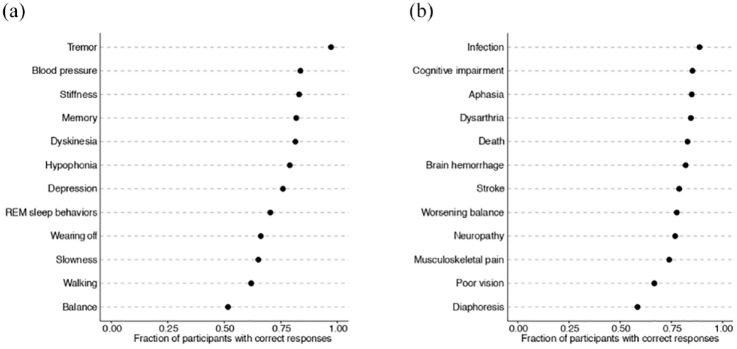
Dot plots demonstrating the fraction of participants (*N* = 77) that correctly identified whether each sign/symptom could be improved by DBS (a) or represented a potential side effect of DBS (b). DBS, deep brain stimulation.

To determine the extent of patient knowledge about DBS, we queried their understanding of the signs and symptoms DBS can be expected to improve [Figure 1(a)] as well as potential side effects associated with DBS [Figure 1(b)]. All questions were answered correctly by at least 50% of participants. No clear trend emerged among motor, non-motor, and medication-related disease manifestations. Tremor, rigidity (stiffness), dyskinesias, blood pressure, and memory constituted the disease impairments most likely to be correctly answered [Figure 1(a)]. On the other hand, diaphoresis, visual acuity, pain, and neuropathy represented the most commonly mis-identified side effects [Figure 1(b)].

We also posed questions regarding the indications for and expectations related to DBS (Table 2). Most patients (53%) considered DBS to be indicated in patients with mid-stage PD, in keeping with 2015 FDA recommendations and interpretation of early PD as ‘a disease duration of more than 6 months but less than 2 years’.^13^ An overwhelming majority acknowledged that medications would continue after the surgical implantation (98.7%). Similarly, they conveyed awareness that DBS requires periodic maintenance (97.4%), including battery replacement (90.9%) and that it has the potential to malfunction (84%).

**Table 2.** Patient perceptions on the timing and expectations for DBS follow-up care.

Statement	*N* = 77^a^
DBS is indicated at the following disease stage:	
Any stage	26 (33.8%)
Early stage	2 (2.6%)
Late stage	6 (7.8%)
Mid stage	40 (52%)
No stage	1 (1.3%)
Meds can be stopped after DBS	1 (1.3%)
DBS requires no maintenance procedures	2 (2.6%)
DBS can malfunction	65 (84%)
DBS battery needs no replacement	7 (9.1%)

*N* reflects the number of patients who answered in the affirmative (yes/true) to the specific statement.

a *n* (%).

DBS, deep brain stimulation.

We subsequently focused on questions or statements that reflected overall judgments of DBS as a positive or negative factor in patient health (Table 3). Eighty-one percent of patients considered DBS at least as effective as the most optimal pharmacological regimen, while 100% of patients agreed that DBS improved their quality of life. Similarly, the majority of patients acknowledged that DBS in select individuals can be superior to treatment with pharmacotherapy alone (93%). Only a small percentage of patients considered DBS to be free of side effects (17%), with 62% perceiving DBS as a disease-modifying therapy.

**Table 3.** Patient outlook on DBS.

Characteristic	*N* = 77^a^
DBS is as effective as meds	62 (81%)
DBS improves quality of life	77 (100%)
DBS has no known side effects	12 (17%)
DBS and meds are superior to meds alone	71 (93%)
DBS can alter clinical course	47 (62%)

*N* denotes the number of patients who answered in the affirmative (yes/true) to the corresponding statement.

a *n* (%).

DBS, deep brain stimulation.

### Determinants of patient knowledge and perceptions of DBS

**Figure 2.**
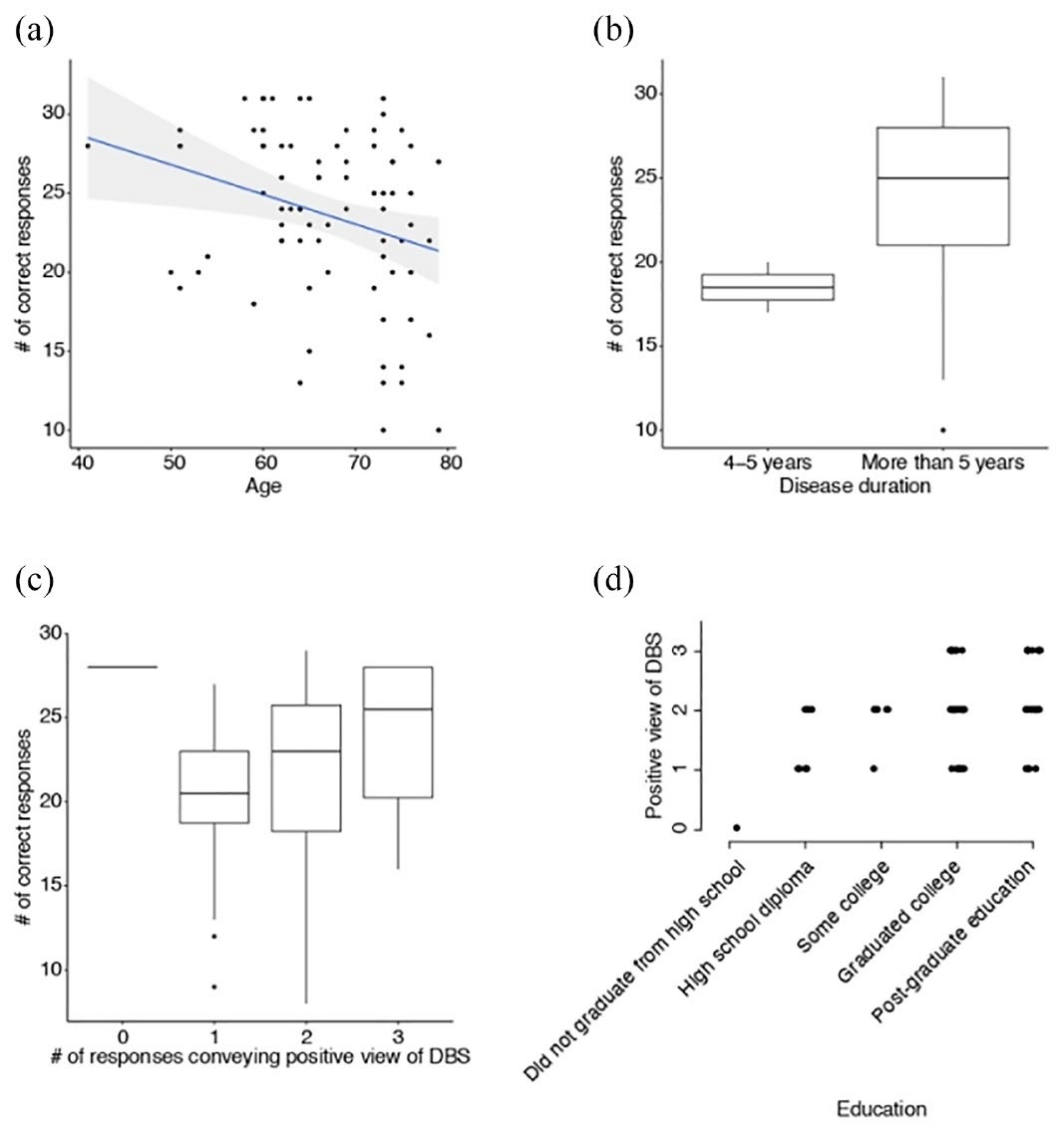
(a) Scatter plot depicting the relationship between age and number of correct responses to factual questions regarding DBS, with superimposed linear regression (model regression coefficient = −0.189, *p* = 0.012). (b) Boxplot demonstrating the distributions of factually correct responses about DBS in patients with a disease duration of less than or greater than 5 years. (c) Boxplot showing the distributions of factually corrected responses regarding DBS stratified by the number of responses conveying a positive outlook of DBS. The boxplot on the left (corresponding to 0 responses conveying a positive outlook) consisted of only one participant. (d) Strip chart showing the distribution of the number of responses conveying a positive outlook of DBS stratified by educational level. Each circle denotes an individual participant. DBS, deep brain stimulation.

To determine the extent to which demographic characteristics underlie patient knowledge of DBS, we modeled the fraction of correct responses to questions discussed in the prior section (pertaining to DBS side effects, indications, and ongoing care) as a function of patient age, disease duration, gender, education level, and race (Methods). The model identified increasing age and a disease duration of greater than 5 years as being associated with lower (OR = 0.96) and higher (OR = 2.17) fraction of correct responses, respectively [Table 4; Figure 2(a) showing the negative slope of a linear fit of the relationship between age and number of correct responses; and Figure 2(b) reflecting the distributions of number of correct responses stratified by disease duration].

**Table 4.** Determinants of patient understanding of DBS.

Characteristic	OR	95% CI	*p* Value
Age	0.96	0.94–0.99	0.001
Gender			
Female	–	–	
Male	0.85	0.57–1.25	0.455
Disease duration			
4–5 years	–	–	
More than 5 years	2.17	0.80–5.81	<0.001
Education			
A few years of college	–	–	
Some education but did not graduate from high school	1.44	0.26–14.0	0.059
High school diploma	1.23	0.51–2.95	0.28
Graduated college	0.96	0.46–1.91	0.740
Post graduate education	0.84	0.39–1.72	0.372
Race			
African American	–	–	
Asian or Pacific Islander	1.08	0.25–4.40	0.860
Caucasian (White)	1.68	0.44–5.94	0.218
Prefer not to say	2.42	0.49–11.9	0.168

Summary statistics (regression coefficients as ORs and statistical significance) of a fractional regression model with fraction of correct responses to factual questions about DBS as the dependent variable and age, gender, disease duration, education, and race as the independent variables. Model *R*^2^ = 0.172.

CI, confidence interval; DBS, deep brain stimulation; OR, odds ratio.

We similarly modeled positive outlook of DBS as a composite of the responses to the questions and statements depicted in Table 3. We omitted the questions pertaining to ‘DBS improvement of quality of life’ and the ‘superiority of DBS and medications over medications alone’ as the responses were practically unanimous and thus would not provide any additional information in distinguishing patients. The model identified education levels up to high school diploma as more strongly associated with lower degrees of positive outlook as reflected in the number of responses to the three questions (Table 5; OR < 1e−8 and OR = 0.41 for education levels at ‘Some education but did not graduate from high school’ and ‘High school diploma’, respectively). This was reflected in the trend of distributions of responses across education level [Figure 2(d)]. Conversely, it also identified the number of factually correct responses as being associated with a greater ‘positive view’ of DBS [Table 5 and Figure 2(c); OR = 1.06].

**Table 5.** Determinants of patient outlook of DBS.

Characteristic	OR	95% CI	*p* Value
Age	1.00	0.97–1.03	0.937
Gender			
Female	–	–	
Male	0.96	0.58–1.59	0.876
Disease duration			
4–5 years	–	–	
More than 5 years	1.86	0.50–6.98	0.350
Education			
A few years of college	–	–	
Some education but did not graduate from high school	0.00^a^		<0.001
High school diploma	0.41	0.13–1.18	0.003
Graduated college	1.00	0.39–2.41	0.996
Post graduate education	1.20	0.45–3.02	0.469
Race			
African American	–	–	
Asian or Pacific Islander	1.36	0.24–7.83	0.582
Caucasian (White)	2.43	0.51–11.9	0.055
Prefer not to say	1.78	0.26–12.7	0.437
Number of correct responses	1.06	1.01–1.11	0.011

Summary statistics (regression coefficients as ORs and statistical significance) of a fractional regression model with fraction of answers conveying a positive outlook of DBS as the dependent variable and age, gender, disease duration, education, race, and patient knowledge (number of correct responses to factual questions related to DBS) as the independent variables. Model *R*^2^ = 0.301.

a Less than 1.0 × 10^−5^.

CI, confidence interval; DBS, deep brain stimulation; OR, odds ratio.

## Discussion

DBS has become a standard therapeutic approach for the management of PD, offering significant motor symptom relief and improving the quality of life of patients. Collectively, the current literature emphasizes the importance of understanding patient perceptions and experiences to optimize DBS outcomes and provide patient-centered care. By addressing factors such as patient expectations, individual variations in response to DBS, and appropriate education, healthcare professionals can better support patients before, during, and after DBS, enhancing overall treatment outcomes and patient satisfaction. Toward this, we sought to provide a comprehensive analysis of patient perceptions of DBS for PD.

Our analysis reflected a strong degree of familiarity with various aspects of the treatment experience. We attribute this to the participant population having been constrained to patients who have been implanted with a DBS device. They would be reasonably more likely to understand the continued role of medications and programming to optimize their symptoms. Similarly, heterogeneity in overall disease burden and treatment response might underlie the spectrum of understanding of both signs and symptoms DBS is intended to improve as well as the side effects.

Demographic variables such as age, disease duration, and education level were statistically positively associated with patient understanding of DBS and a positive outlook of it. However, it is important to note that this study has limited generalizability as most participants were White, highly educated, and had longer disease duration. Moreover, we did not specifically ask about disease severity, functional impact of DBS, extent of pre- and post-implantation counseling, mental health, caregiver burden, and time since DBS implantation, all of which could impact on knowledge and outlook. For example, cognitive impairment, which also independently correlates with age,^14^ might partially explain the weak association between increasing age and patient knowledge. On the other hand, disease severity is unlikely to segregate with educational level in the same vein as suboptimal implantation is likely largely independent of many of the demographic variables tested. All of these considerations directly motivate future investigations into more causal links between demographic variables such as age and education and patient experience. This is all the more important given our observations that patients with greater knowledge of DBS and higher education level are more likely to hold a positive perspective of DBS.

## Conclusion

Our research offers a comprehensive insight into how patients perceive DBS as a treatment for PD, encompassing its advantages, difficulties, and misunderstandings. Understanding such disparities would allow clinicians to tailor counseling related to DBS to optimize postoperative outcomes and address realistic and unrealistic expectations of individual patients prior to DBS in greater detail.

## Declarations

**Ethics approval and consent to participate:** The study was approved by the UT Southwestern Institutional Review Board (#STU-2022-0250), with informed consent provided by voluntarily accessing a link to the survey. We confirm that we have read the Journal’s position on issues involved in ethical publication and affirm that this work is consistent with those guidelines.

**Consent for publication:** Not applicable.

**Availability of data and materials:** Data is available upon reasonable request to corresponding author.
